# Hawaiʻi Coral Disease database (HICORDIS): species-specific coral health data from across the Hawaiian archipelago

**DOI:** 10.1016/j.dib.2016.07.025

**Published:** 2016-07-19

**Authors:** Jamie M. Caldwell, John H.R. Burns, Courtney Couch, Megan Ross, Christina Runyon, Misaki Takabayashi, Bernardo Vargas-Ángel, William Walsh, Maya Walton, Darla White, Gareth Williams, Scott F. Heron

**Affiliations:** a Hawaiʻi Institute of Marine Biology, School of Ocean and Earth Science and Technology, University of Hawaiʻi, Kāneʻohe, HI 96744, USA; b Department of Microbiology, University of Hawaiʻi at Mānoa, Honolulu, HI 96822, USA; c Marine Science Department, University of Hawaiʻi, Hilo, HI 96720, USA; d Joint Institute for Marine and Atmospheric Research, University of Hawaiʻi, Honolulu, HI 96822, USA; e Hawaiʻi Division of Aquatic Resources, Kailua-Kona, HI 96740, USA; f University of Hawaiʻi Sea Grant College Program, Honolulu, HI 96822, USA; g Maui Division of Aquatic Resources, Wailuku, HI 96793, USA; h School of Ocean Sciences, Bangor University, Anglesey LL59 5AB, United Kingdom; i Coral Reef Watch, U.S. National Oceanic and Atmospheric Administration, College Park, MD 20740, USA; j Marine Geophysical Laboratory, Physics Department, College of Science, Technology and Engineering, James Cook University, Townsville, QLD 4811, Australia; k Global Science and Technology, Inc., Greenbelt, MD 20770, USA

**Keywords:** Marine biology, Coral, Reefs, Disease, Hawaii

## Abstract

The Hawaiʻi Coral Disease database (HICORDIS) houses data on colony-level coral health condition observed across the Hawaiian archipelago, providing information to conduct future analyses on coral reef health in an era of changing environmental conditions. Colonies were identified to the lowest taxonomic classification possible (species or genera), measured and assessed for visual signs of health condition. Data were recorded for 286,071 coral colonies surveyed on 1819 transects at 660 sites between 2005 and 2015. The database contains observations for 60 species from 22 genera with 21 different health conditions. The goals of the HICORDIS database are to: i) provide open access, quality controlled and validated coral health data assembled from disparate surveys conducted across Hawaiʻi; ii) facilitate appropriate crediting of data; and iii) encourage future analyses of coral reef health. In this article, we describe and provide data from the HICORDIS database. The data presented in this paper were used in the research article “Satellite SST-based Coral Disease Outbreak Predictions for the Hawaiian Archipelago” (Caldwell et al., 2016) [1].

**Specifications Table**

**Table:** 

Subject area	*Biology*
More specific subject area	*Marine ecology*
Type of data	*Table, figure*
How data was acquired	*Underwater visual surveys conducted on SCUBA and snorkel*
Data format	*Raw*
Experimental factors	*286,071 coral colonies observed from 17 Hawaiian islands and atolls between 2005 and 2015*
Experimental features	*Species identification, colony measurements, health condition, GPS coordinates and depth*
Data source location	*Hawaiian archipelago extending from Hawai׳i Island to Kure Atoll*
Data accessibility	*Data available within this article* (Table S1)

**Value of the data**

• The data can be used to analyze coral community composition and relationships between community composition and environment;
• Compare spatial and temporal trends in coral disease severity and prevalence in Hawaiʻi;
• Investigate the role of trait-based and environmental factors contributing to disease presence and/or severity;
• Compare disease patterns in Hawaiʻi with observations from different regions around the world;
• Create new and more accurate forecasting models of disease outbreaks.

## 1 Data

**Fig. 1:**
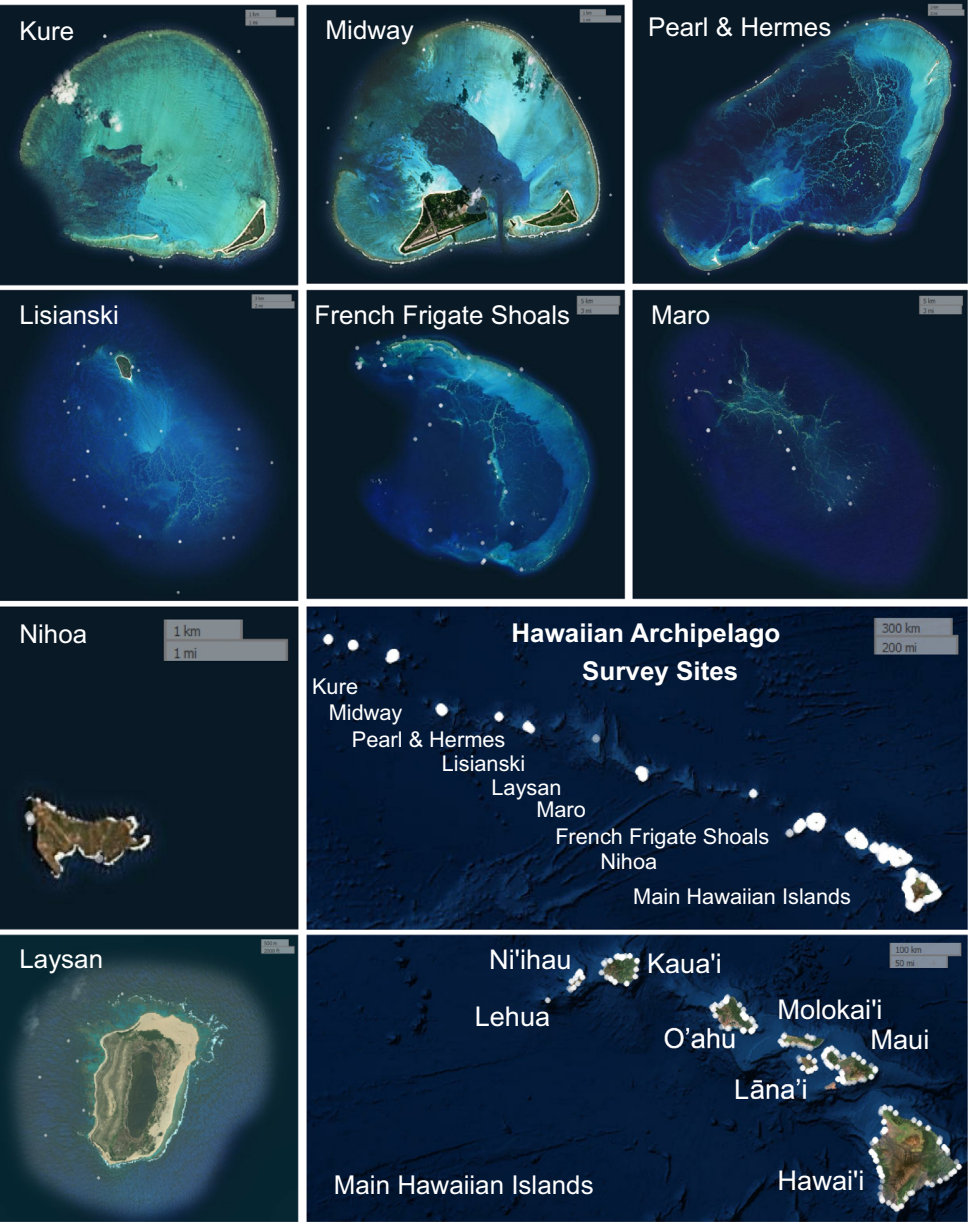
Map of survey locations in the Hawaiian archipelago. White dots indicate survey locations.

The Hawaiʻi Coral Disease database (HICORDIS) consists of observational surveys of coral health conducted across the Hawaiian archipelago between 2005 and 2015 (Table S1). Ten research groups from academic institutions (University of Hawaiʻi at Mānoa, University of Hawaiʻi, Hilo, Cornell University, University of Wellington), state and federal agencies (Hawaiʻi/Maui Division of Aquatic Resources, National Oceanic and Atmospheric Administration Coral Reef Ecosystem Program) collected survey data at 17 islands and atolls spanning nine degrees of latitude (Fig. 1, Table S2). Data were recorded for 286,071 coral colonies on 1819 transects at 660 sites (Fig. 1). These sites capture the variability in coral community composition and environmental conditions that occur across the ~2400 km Hawaiian archipelago. The data presented in this paper were used in the research article “Satellite SST-based Coral Disease Outbreak Predictions for the Hawaiian Archipelago” [1].

## 2 Experimental design, materials and methods

### 2.1 Survey methods

Observations of coral colony health were collected by one of three survey techniques: belt transects with direct or indirect measures of prevalence or line-intercept. For the belt transect method with direct measures of prevalence, divers recorded every coral colony׳s health condition within a specified area (average length=20 m, range=8–50 m; average width=1 m, range=1–6 m). In the belt transect method with indirect measures of disease prevalence, divers counted all colonies with a health condition (e.g., disease) within a large belt transect area (average area=25×2 m^2^, range=25×2 m^2^ to 25×6 m^2^) and counted the total number of coral colonies in a subset region of the large belt transect area (average area of subset region=10×2 m^2^, range=10×2 m^2^ to 10×6 m^2^). In the line-intercept method, divers recorded coral health state for every colony directly under 25 m of transect tape. Survey depths ranged from <1 to 26 m.

### 2.2 Coral colony data

All coral colonies in the HICORDIS database were classified taxonomically and visually assessed for coral health conditions. Observations were recorded for 60 coral species from 22 genera (Table S3). There were 21 possible classifications for coral health state (Tables S4 and S5). Health classifications included no visible lesions, known coral diseases, bleaching, discoloration patterns, algal and bacterial infections and predatory invertebrates. In total, 17% of coral colonies exhibited visual signs of compromised health conditions (Table S4). When data was available, disease severity measurements were also incorporated. Disease severity was quantified as the percent of live tissue affected by a health condition. We note that for coral bleaching in particular, the severity metric we provide here may not be the best measure of severity (e.g., categorizing severity as pale, mottled or stark white may be more indicative of bleaching severity than amount of surface tissue affected).

**Fig. 2:**
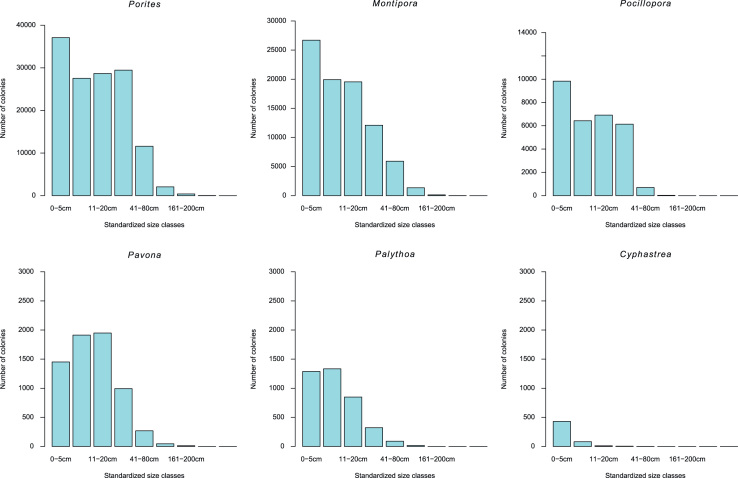
Size frequency distributions for the six most common coral genera recorded in the HICORDIS database.

**Fig. 3:**
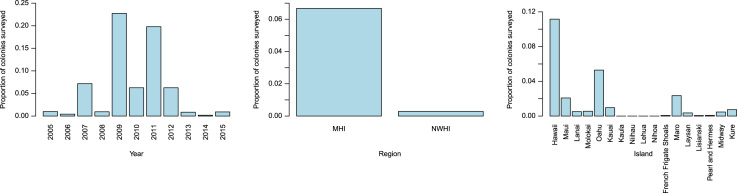
Variation in *Porites* growth anomalies by year (left), region (center) and island (right).

Most records in the HICORDIS database included a measurement of coral colony size. There were up to four types of measurements recorded for each colony: colony length, colony width, size classes and standardized size classes. Colony lengths and widths were measured as the two longest horizontal axes along a coral colony. Colony lengths ranged from <1 to 410 cm; colony widths ranged from <1 to 190 cm. Size classes were used to bin colonies into size ranges; however, size classes varied among research groups. In order to facilitate comparisons among observations, a “standardized size class” was included, which grouped coral colonies into size bins based on colony length, colony width and/or size class. Standardized size classes consist of the following size bins: 0–5 cm; 6–10 cm; 11–20 cm; 21–80 cm; 81–160 cm; 161-200 cm; 201-300 cm; 301-450 cm (Fig. 2). An example of how this data can be used to compare coral health across size classes, space and time is provided in Fig. 3. All missing data in the HICRODIS database was recorded as “NA”.

## Appendix A Supplementary material

Supplementary material **Table S1: HICORDIS data**.

Supplementary material **Table S2: Number of colonies surveyed at each island/atoll by year. FFS: French Frigate Shoals; PHR: Pearl and Hermes Reef. Hyphens indicate no surveys were conducted**.

Supplementary material **Table S3: Genus and species recorded in the HICORDIS database. Zoanthus was only recorded to genus**.

Supplementary material **Table S4: Frequency of colonies observed with each health condition. Health conditions are listed in descending order based on the frequency of observations**.

Supplementary material **Table S5: Description of coral health conditions in the HICORDIS database**.
